# Lower Extremity Arterial Occlusive Disease and Abdominal Aortic Aneurysm in Crohn’s Disease: A Case Report

**DOI:** 10.7759/cureus.103675

**Published:** 2026-02-15

**Authors:** Yohei Yamamoto, Tsuyoshi Ichinose, Toshifumi Kudo

**Affiliations:** 1 Division of Vascular Surgery, Department of Cardiovascular Surgery, Institute of Science Tokyo, Tokyo, JPN

**Keywords:** abdominal aortic aneurysm, crohn’s disease, endovascular aneurysm repair, extraintestinal manifestation, lower extremity arterial disease

## Abstract

Crohn’s disease (CD) is a type of inflammatory bowel disease that primarily affects the small intestine and colon. Patients with CD may develop extra-intestinal manifestations; however, vascular involvement has been rarely reported. Herein, we present the case of a patient with CD who developed multiple arterial complications, including lower extremity arterial occlusions and an abdominal aortic aneurysm (AAA).

A 42-year-old man with CD presented with back pain. He also had a history of bilateral iliac artery occlusions that were refractory to revascularization procedures. Enhanced computed tomography revealed an irregularly shaped AAA with a maximum axial diameter of 72 × 52 mm. The patient underwent endovascular aneurysm repair (EVAR) for the AAA via the left axillary access, and the postoperative course was uneventful. At seven years postoperatively, significant shrinkage of the aneurysm was maintained, and his CD remained in remission.

The present case suggests that patients with CD can develop AAA as a rare extra-intestinal manifestation. Significant aneurysm shrinkage was achieved following EVAR.

## Introduction

Crohn’s disease (CD) is a type of inflammatory bowel disease (IBD) that primarily affects the small intestine and colon [1]. It is characterized by transmural inflammation, which can lead to complications, including strictures and fistulas. Patients with CD may develop extra-intestinal manifestations, including cardiovascular complications [2].

Arterial and venous thrombosis are common cardiovascular complications; however, reports of other vascular manifestations are limited. Herein, we present the case of a patient with CD who developed an abdominal aortic aneurysm (AAA) following lower extremity arterial occlusions. The patient consented to the publication of this case report.

## Case presentation

A 42-year-old man presented with back pain. His medical history was notable for CD, which was diagnosed at the age of 30. He underwent an ileocecal resection for ileal stenosis. At the age of 35, he developed occlusion of the bilateral iliac arteries. Although the details are unknown, he underwent multiple revascularization attempts, including a femoro-femoral crossover bypass, all of which ultimately failed. His symptom was mild intermittent claudication, which did not progress to limb-threatening ischemia; therefore, he was managed with medical treatment and followed up regularly. For several years leading up to this presentation, the patient was treated with 3 g/day of mesalazine and 200 mg/day of cilostazol, and his disease remained stable. Aside from CD and lower extremity arterial occlusions, he had no other significant medical history. He had a smoking history of 20 cigarettes per day from the age of 20 to 35. On physical examination, a pulsatile abdominal mass was palpated. His bilateral femoral pulses were absent, and the ankle-brachial pressure index was 0.57 in the right leg and 0.61 in the left. The patient reported no recent history of fever. Laboratory findings on presentation showed a white blood cell count of 7.6 × 10⁹/L, a hemoglobin level of 11.4 g/dL, and a C-reactive protein level of 1.93 mg/dL (Table 1).

**Table 1:** Laboratory data on presentation

Valuables	Results (reference range)
White blood cell count (×10^9^/L)	7.6 (3.3-8.6)
Hemoglobin (g/dL)	11.4 (13.7-16.8)
Platelet count (×10^4^/μL)	28.8 (15.8-34.8)
Erythrocyte sedimentation rate (mm/h)	21 (2-10)
C-reactive protein (mg/dL)	1.93 (0.00-0.14)
Serum albumin (g/dL)	3.3 (4.1-5.1)
Serum creatinine (mg/dL)	0.82 (0.65-1.07)
Serum total cholesterol (mg/dL)	90 (142-248)
D-dimer (μg/mL)	4.97 (0.00-0.99)

**Figure 1:**
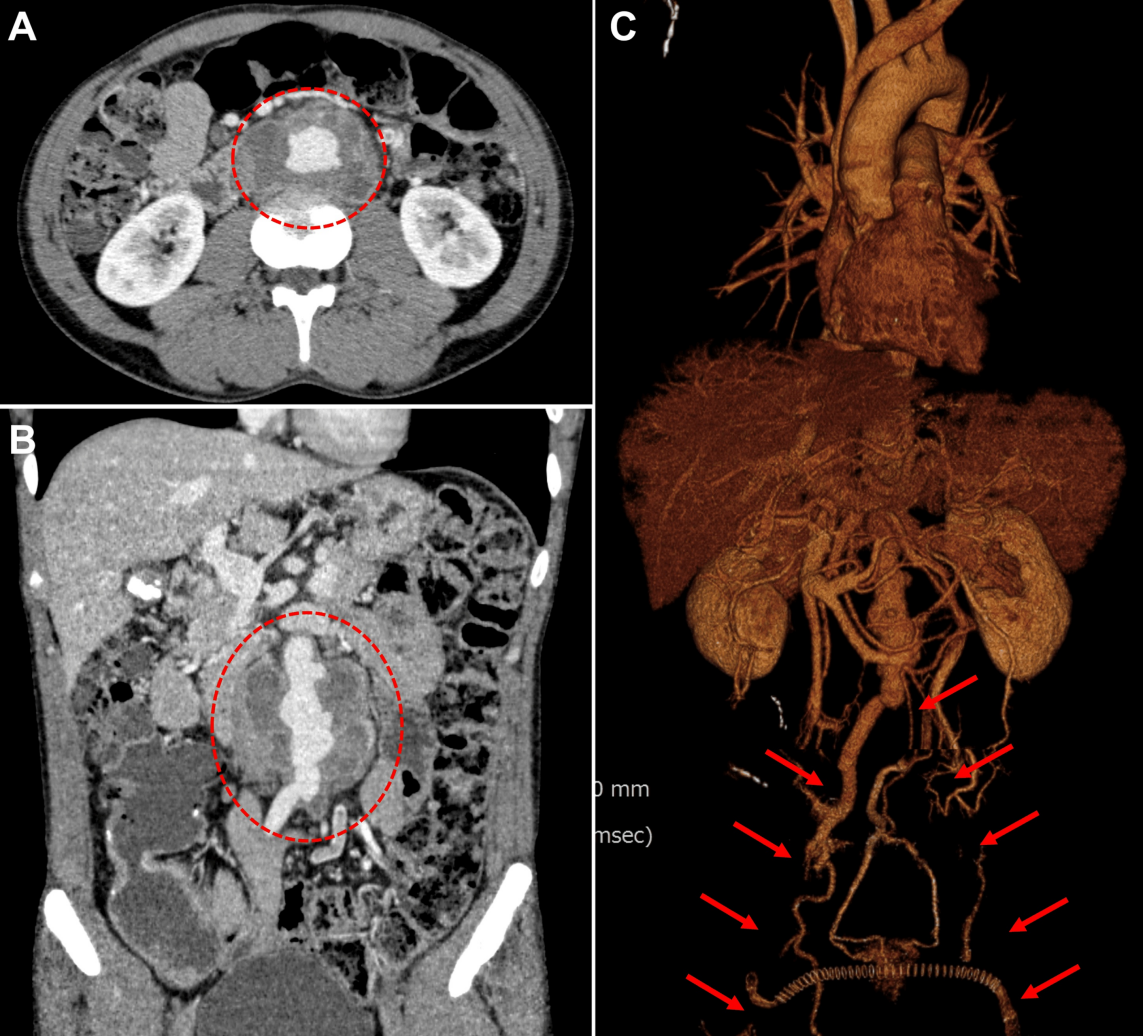
Preoperative computed tomography images (A) Axial, (B) coronal, and (C) three-dimensional images showing an irregularly shaped AAA (circles) and occluded bilateral lower extremity arteries (arrows). AAA: abdominal aortic aneurysm

Enhanced computed tomography revealed an irregularly shaped AAA with a maximum axial diameter of 72 × 52 mm, along with known lower extremity occlusive lesions (Figure 1).

**Figure 2:**
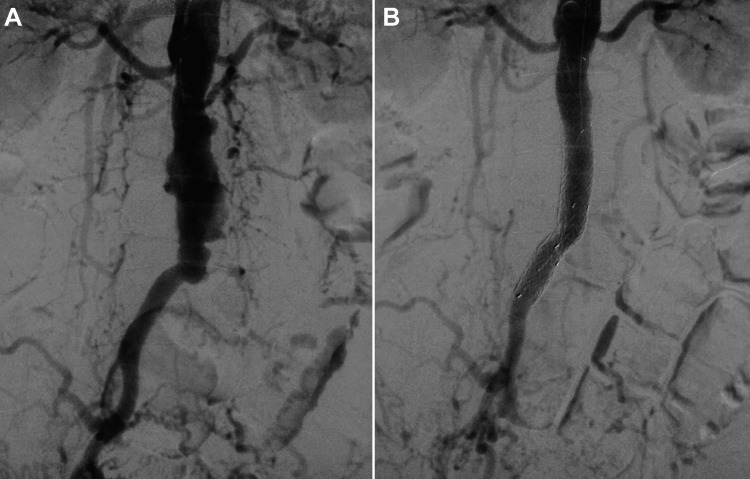
Intraoperative angiogram of the aorta (A) Initial angiogram and (B) completion angiogram after stentgraft deployment.

On a computed tomography scan performed 21 months earlier, mild wall thickening of the infrarenal aorta was noted; however, no aneurysmal dilatation was identified. Additionally, a colonoscopy was performed to assess CD activity, which revealed an anastomotic ulcer. Initial and repeat blood cultures were negative. Given the aneurysm’s size and its possible association with his back pain, aneurysm repair was indicated. The length of the non-aneurysmal infrarenal aortic neck was approximately 16 mm, and the right common iliac artery was deemed suitable for distal sealing. The orifice of the inferior mesenteric artery was occluded. Considering his history of multiple laparotomies, endovascular aneurysm repair (EVAR) was deemed preferable. As both iliac arteries were occluded, left axillary access was selected and surgically exposed via a cut-down approach. EVAR was then performed using two straight stent grafts (Gore Excluder PLC161000J and PLC201200J; W.L. Gore & Associates Inc., Flagstaff, AZ, USA) through this access (Figure 2).

**Figure 3:**
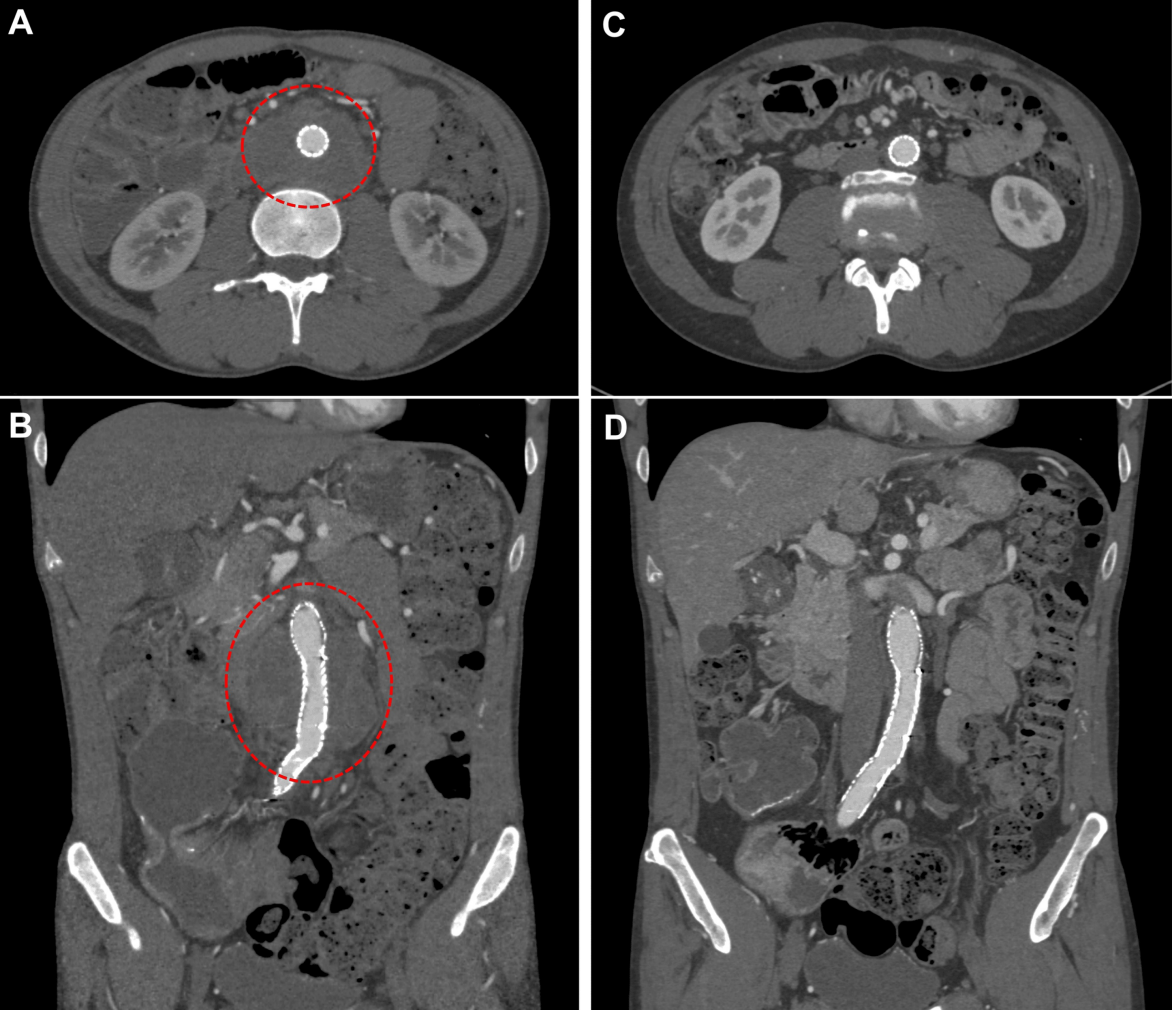
Postoperative computed tomography images Images obtained (A, B) at one week and (C, D) at five years after surgery, showing shrinkage of the aneurysm. The circles indicate the aneurysmal sac.

Intraoperative angiograms revealed abundant collateral blood flow from the right internal iliac artery to the bilateral lower extremities. Although the developed lumbar arteries were covered by the stent grafts, bilateral ankle blood pressures were unchanged after stent graft placement. Therefore, additional revascularization of the lower extremities was not performed. The postoperative course was uneventful, and he was discharged eight days after surgery. Postoperatively, he commenced additional immunosuppressive therapy with oral prednisolone, initially at 65 mg/day, which was gradually tapered to 5 mg/day, and azathioprine at 25 mg/day. Postoperative imaging follow-up using computed tomography or duplex ultrasound was performed at one week, three months, six months, and one year after EVAR, and annually thereafter. Postoperative follow-up computed tomography scans performed five years after surgery demonstrated significant aneurysm shrinkage (Figure 3).

At seven years postoperatively, significant shrinkage of the aneurysm was maintained, and his CD remained in remission.

## Discussion

In this case report, we present a patient with CD who developed multiple arterial complications, including an AAA. CD is a type of IBD that mainly affects the small intestine and the colon [1].

Cardiovascular complications are among the important extra-intestinal manifestations in patients with IBD [2], and thromboembolism is the most common vascular manifestation. A large early study reported that 1.3% of patients with IBD developed arterial or venous thrombosis over 10 years, representing the incidence of these events [3]. Venous thromboembolism is more frequent than arterial events. Studies conducted around 2000 demonstrated that the prevalence of venous thromboembolism in patients with IBD was approximately 6%, with a three-fold increase in risk compared to the general population [4,5].

Some reports suggest that patients with IBD, especially young patients with CD, have an increased risk of atherosclerotic cardiovascular disease and lower extremity arterial occlusions [6,7]. Our patient developed bilateral lower extremity arterial occlusions five years after the diagnosis of CD, at the age of 35. Similarly, several reports have documented cases of CD with bilateral iliac artery occlusion [7,8]. In these instances, premature atherosclerosis [7] or arterial wall inflammation [8] is considered the cause of arterial occlusions.

The most remarkable finding in our patient was the development of an irregularly shaped AAA following refractory lower extremity occlusive disease. Yang et al. reported that patients with ulcerative colitis have an approximately threefold increased risk of developing AAA compared with propensity score-matched controls [9]. However, to the best of our knowledge, the incidence of AAA in patients with CD has not been previously reported. Although histologic confirmation is lacking, the patient's clinical course and the aneurysm's irregular configuration suggest an inflammatory etiology. Additionally, increased aortic pressure due to occlusion of the bilateral iliac arteries may have contributed to the aneurysm’s progression. The preoperative findings did not conclusively rule out an infectious etiology; however, repeated negative blood cultures during the clinical course, together with a favorable postoperative course without signs of infection, were considered suggestive of a noninfectious etiology.

Several cases of abdominal aortitis have been reported in patients with CD [10-12]. CD is recognized as a systemic inflammatory disorder, and chronic immune activation and shared genetic risk factors may contribute not only to a prothrombotic state but also to large-vessel inflammation, such as aortitis [2]. CD has also been associated with Takayasu arteritis [13]. Therefore, it is essential to exclude Takayasu arteritis in patients with CD suspected of aortitis. The coexistence of CD and Takayasu arteritis suggests a potential shared autoimmune mechanism [14]. In the present case, the diagnostic criteria for Takayasu arteritis were not fulfilled because the aortic arch and its branches were normal.

Symptomatic AAAs should be treated by conventional open repair or EVAR, regardless of their etiology. In the present case, treatment was performed under the suspicion of an inflammatory aneurysm. In the management of inflammatory aneurysms, open repair is associated with a lower incidence of persistent peri-aneurysmal inflammation and related complications during follow-up. Conversely, EVAR is associated with lower 30-day mortality and fewer iatrogenic injuries than open repair [15]. Given that our patient had undergone multiple laparotomies, we opted for EVAR. Notably, significant aneurysm shrinkage was achieved postoperatively. This significant postoperative negative remodeling may be due to decreased wall tension by the stent graft and reduced inflammation in the aortic wall by the newly added immunosuppressive therapy.

## Conclusions

This case suggests that patients with CD may present with multiple arterial complications, including AAA, as rare extra-intestinal vascular manifestations. EVAR, followed by additional immunosuppressive therapy, was feasible in this patient, and significant aneurysm shrinkage was observed during long-term follow-up. Although a causal relationship cannot be established from a single case, clinicians should be aware of the potential for vascular involvement in patients with CD. They should consider appropriate imaging and individualized management during follow-up.
